# Evaluation of apoptosis stimulating protein of TP53-1 (ASPP1/*PPP1R13B*) to predict therapy resistance and overall survival in acute myeloid leukemia (AML)

**DOI:** 10.1038/s41419-023-06372-0

**Published:** 2024-01-10

**Authors:** Marcus M. Schittenhelm, Max Kaiser, Balázs Győrffy, Kerstin M. Kampa-Schittenhelm

**Affiliations:** 1 Medical research center (MFZ) and Clinic of Medical Oncology and Hematology, Cantonal Hospital St. Gallen (KSSG), St. Gallen, Switzerland; 2 Department of Hematology, Oncology, Clinical Immunology and Rheumatology, University Hospital Tübingen (UKT), Tübingen, Germany; 3 Semmelweis University Dept. of Bioinformatics and Dept. of Pediatrics, Budapest, H-1094 Hungary; 4 TTK Cancer Biomarker Research Group, Institute of Enzymology, Budapest, H-1117 Hungary

**Keywords:** Acute myeloid leukaemia, Apoptosis, Prognostic markers

## Abstract

ASPP1 (*PPP1R13B*) belongs to a family of p53-binding proteins and enhances apoptosis by stimulation of p53-transactivation of selected proapoptotic target genes. It is preferentially expressed in hematopoietic stem cells (HSC) and together with p53 preserves the genomic integrity of the HSC pool. Consequently, dysfunction of ASPP1 has been associated with malignant transformation and development of acute lymphoblastic leukemias and lymphomas - whereas methylation of the promoter region is linked to reduced transcription and ultimately attenuated expression of ASPP1. The role of ASPP1 in AML is not known. We now show that impaired regulation of *PPP1R13B* contributes to the biology of leukemogenesis and primary therapy resistance in AML. *PPP1R13B* mRNA expression patterns thereby define a distinct prognostic profile - which is not reflected by the European leukemia net (ELN) risk score. These findings have direct therapeutic implications and we provide a strategy to restore ASPP1 protein levels using hypomethylating agents to sensitize cells towards proapoptotic drugs. Prospective clinical trials are warranted to investigate the role of ASPP1 *(PPP1R13B)* as a biomarker for risk stratification and as a potential therapeutic target to restore susceptibility to chemotherapy.

## Introduction

The tumor suppressor p53 (*TP53*) is well known for its key role in cellular stress response and prevention of tumor formation. Not surprisingly, inactivation of the p53 pathway is a frequent event in human cancer and *TP53* represents one of the most frequently mutated genes with over 50% of human malignancies harboring inactivating *TP53* mutations [1, 2].

Intriguingly, in de novo acute myeloid leukemia (AML) inactivating *TP53* mutations or chromosomal aberrations of the *TP53* gene locus are uncommon [3]. We therefore hypothesized that disruption of the p53 pathway in AML is mediated by other mechanisms.

We have evidence that dysregulation of members of the Apoptosis Stimulating Protein of p53 (ASPP) protein family are involved in this process. Three members of the ASPP family exist: ASPP1 and ASPP2 bind to the p53 core domain and thereby enhance DNA binding and transactivation function of p53 on the promoters of selected pro-apoptotic target genes in vivo (reviewed by Sullivan et al. [4]). Inhibitory-(i)ASPP acts as an inhibitor of apoptosis by binding to an adjacent region of the core domain and the proline rich region of *TP53* [5].

In recent studies, ASPP2 was identified as a haploinsufficient tumor suppressor [6]. Further, we demonstrated that mRNA and consequently protein levels of ASPP2 are frequently attenuated in AML, presumably by methylation of the promoter and untranslated transcription start site (UTS). Lack of ASPP2 expression was thereby highly associated with therapy failure to induction chemotherapy [7].

The role of ASPP1 as a tumor suppressor in mouse models is less clear [8], however it was shown that ASPP1 preserves the genomic integrity of murine hematopoietic stem cells in vivo and thereby, together with p53, prevents malignant transformation [9].

Further, attenuated expression levels of ASPP1 have been described in patients suffering from acute lymphoblastic leukemia (ALL) – which again was linked to methylation of the *PPP1R13B* promoter region [10].

In addition, ASPP1 has p53-independent functions to control induction of apoptosis, such as via p63 and p73 [11] – or even counteracting antiapoptotic functions depending on the subcellular localization of ASPP1: A yeast two-hybrid screen identified cytoplasmatic ASPP1 as a binding partner of Yes-associated protein (YEP) to inhibit apoptosis [12]. These findings put ASPP1 in a complex and central role to control apoptosis.

The role of ASPP1 (*PPP1R13B*) in AML is not known – and is addressed herein. We will show that expression of *PPP1R13B* mRNA is frequently attenuated in AML, which has functional and prognostic consequences in defined patient cohorts. These findings will have clinically meaningful implications to guide therapy in AML.

## Methods

### Isolation of bone marrow and peripheral blood mononuclear cells

Bone marrow aspirate and peripheral blood samples from patients with diagnosed acute myeloid leukemia or healthy volunteers (bone marrow or blood donors) were collected in 5 000 U heparin after written informed consent and approval of the ethics committee of the University of Tübingen (188/2018BO2). Mononuclear cells were isolated by Ficoll Hypaque density gradient fractionation.

### Polymerase chain reaction (PCR)

mRNA was isolated and reversely transcribed using standard techniques and a RNeasy® RNA purification kit (Qiagen, Hilden, Germany). *PPP1R13B* mRNA expression levels, relative to *GAPDH* as a housekeeping gene, were determined by qRT-PCR Roche® LightCycler Technology (Roche, Basel, Switzerland). The *PPP1R13B* primer set was as follows: sense 5‘-TCATGAACGGCAATCTGTCT-3‘), antisense 5‘-GATCAACCCTTAAAATTGCAGTC-3‘).

### Statistical analysis

Unselected patient cohort: An unselected patient cohort of newly diagnosed AML (*n* = 39, patient characteristics are provided in Table 1) and healthy volunteers (*n* = 12) were assessed according to subcohorts using the Student’s *t* test and the Mann–Whitney U test for parametric and nonparametric datasets, respectively. Spearman’s rank correlation coefficient was used to assess significance of clinical parameters according to ASPP1 (*PPP1R13B* mRNA) expression levels.

**Table 1:** Patient characteristics.

*Secondary AML* defined as *AML* progressing from MDS or MDS/MPN or post-cytotoxic therapy [13], *WBC* white blood cell count, *R* responder, *NR* non-responder, *NA* not applicable (no induction therapy).

#### RNAseq dataset

In the RNAseq analysis, a robust and comprehensive dataset of an integrated database of highly regarded sources (as explained in reference [13]) was compiled. In particular, these sources include three key repositories: the Cancer Genome Atlas (TCGA), the Therapeutically Applicable Research to Generate Effective Treatments (TARGET) database, and the Genotype-Tissue Expression (GTEx) database of normal tissue. The combination of the three databases creates a large and comprehensive dataset comprising *n* = 56,938 samples. This substantial sample size is critical to generating robust and meaningful conclusions.

The Kaplan–Meier method was used for the survival analysis. To perform this analysis, we used the KM Plotter web platform, a powerful computational tool available at KMplot.com/private edition. This platform allows the examination of survival data based on differential expression characteristics.

#### Transcriptome dataset

The data set derives from a gene expression omnibus (GEO) repository with published follow-up data and mutation status for a selected set of established prognostic genes, useful to assess for the correlation between *PPP1R13B* expression levels and survival data (GEO accession numbers GSE1159 [14] and GSE6891) [15].

The datasets include all together 830 patient samples, average age 41.7 years, 49.4% male (*n* = 349, of 708 with known gender), and 50.0 months average follow-up with 66.6% of the patients having an event (*n* = 520). Promyelocytic leukemia cases with FAB M3 morphology were excluded.

We employed Cox proportional hazards regression to assess differential survival outcomes. To mitigate potential oversight of correlations attributed to a specific threshold, we exhaustively considered all available cut-off values falling within the lower and upper quartiles of gene expression. We applied the Benjamini–Hochberg method to estimate the false discovery rate (FDR) and account for multiple hypothesis testing. The cut-off value yielding the highest statistical significance (minimizing FDR) was identified. In instances where multiple cut-off values exhibited identical significance, we prioritized the cut-off with the highest hazard rate (HR) for the subsequent analysis. Subsequently, we generated Kaplan–Meier plots to visually represent survival disparities, utilizing the determined cut-off values from the aforementioned analysis.

### Cell lines

The acute myeloid leukemia cell line MOLM14 was obtained from DSMZ (No.: ACC 777). Cells were cultured in RPMI 1640, supplemented with 10% fetal bovine serum, 1% penicillin G (10 000 units/mL) and streptomycin (10,000 µg/mg) and 2 mmol/L l-glutamine (GIBCO/Invitrogen, Darmstadt, Germany or Biochrom AG, Berlin, Germany).

### Antibodies and reagents

An anti-ASPP1 monoclonal mouse antibody, clone LXO54.2, was obtained from Sigma-Aldrich (A4355). Anti-tubulin antibody was used as a loading control (#2144, Cell Signaling, Danvers, MA).

Decitabine, cytarabine and daunorubicin were obtained from the University of Tübingen Hospital Pharmacy and dissolved in DMSO to stock solutions and stored at −20 °C.

### Short hairpin RNA (shRNA)-interference

For specific suppression of *PPP1R13B* mRNA transcription an shRNA approach was established. A GIPZ lentiviral shRNAmir system from Dharmacon (Lafayette, CO) was used according to the manufacturers protocol. In short, after plasmid replication and production of lentiviral particles in a HEK293T cell line, sh*PPP1R13B*_MOLM14 leukemia cell strains were established using puromycin selection to stably suppress *PPP1R13B* mRNA transcription. Empty vector (EV) constructs served as negative control strains. TurboGFP reporter was used to assess for transduction efficiency in a flow cytometer. Knock down of ASPP1 (*PPP1R13B* mRNA*)* expression was verified on the mRNA (qRT-PCR) as well as protein level (immunoblot). PCR assay was performed in quintuplets per run.

### Western immunoblot

Immunoblotting was performed as previously described [16] using SDS-PAGE and wet transfer on nitrocellulose membranes. Membranes were blocked in 10% BSA solution. An infrared imaging system (Li-COR Odyssey, Lincoln, NE) was used for quantitative protein visualization.

### Apoptosis assay

For the detection of early and late apoptotic cells an annexin V/propidium iodide (PI)-based flow cytometry assay was used according to the manufacturers protocol and as previously described [16]. IC50s were calculated using a dose-response curve generated with GraphPad Prism® software (Boston, MA). Experiments were performed at least in technical triplicates.

### Proliferation assay

To determine the proliferation capacity cell numbers were subsequently counted for 4 days at fixed 24-hour intervals using a Neubauer Counting Chamber. Assuming an exponential growth, formula 1 was used to determine the growth constant λ. Cell doubling time td was determined using formula 2.

$$\begin{array}{ll}{\rm{Formula}}\,1 & {\rm{Formula}}\,2\\ {\lambda }=\frac{{\rm{In}}({N}_{t})-{\rm{In}}({N}_{0})}{t-{t}_{0}} & {t}_{d}=\frac{{\rm{In}}(2)}{{\lambda }}\end{array}$$

t0 = initial time, t = observation time, N0 = initial cell count, Nt = cell number at time t, λ = growth constant, td = doubling time

Experiments were performed at least in technical triplicates.

In addition, an XTT-based viability assay was used according to the manufactures protocol to determine proliferation capacity – whereas the measured absorption rates correlate with the number of metabolically active cells [17]. Assay was performed *n* = 3 with technical repeats *n* = 5 per run.

## Results

### *PPP1R13B* mRNA expression is significantly attenuated in acute myeloid leukemia

mRNA expression patterns of *PPP1R13B* was assessed in an unselected patient cohort with newly diagnosed AML (*n* = 39, patient characteristics provided in Table 1). Peripheral blood (pb) as well as bone marrow (bm) aspirates were available for this analysis - whereas no significant differences in *PPP1R13B* expression levels were detected between pb and bm samples (data not shown, *p* = 0.60).

**Fig. 1:**
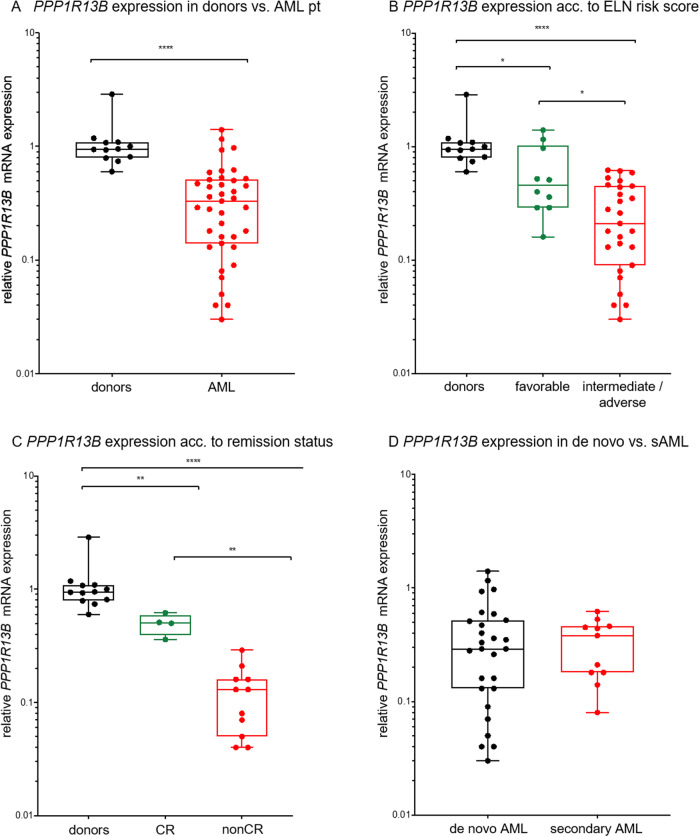
qRT-PCR based mRNA expression of *PPP1R13B* in AML. *PPP1R13B* expression (**A**) in an unselected AML pt. population vs. healthy bone marrow donor cohort, (**B**) according to ELN 2017 risk categories, (**C**) according to response to induction chemotherapy and (**D**) in de novo vs. secondary AML (defined as AML progressing from MDS or MDS/MPN or post-cytotoxic therapy). ns not significant, *p < 0.05, **p < 0.01, ***p < 0.001, ****p < 0.0001 (Mann–Whitney test).

Comparison of expression levels in AML vs. bone marrow aspirates of 12 healthy donors demonstrated significantly attenuated relative median expression levels of *PPP1R13B* in the AML cohort (0.33 (*PPP1R13B*_AML_) vs. 0.95 (*PPP1R13B*_donor_), *p* < 0.0001), Fig. 1A.

Interestingly, *PPP1R13B* mRNA expression showed wide dispersion in the AML cases with relative expression levels ranging from a minimum of 0.03 to a maximum of 1.4. Thus, subcohort analysis was performed to evaluate whether *PPP1R13B* mRNA expression links to a specific risk profile: 37 patients with known molecular characteristics were categorized according to the European LeukemiaNet (ELN) 2017 genetic risk classification [18], which was the current risk stratification system at the time when patients were treated.

Indeed, comparing favorable risk vs. higher risk patient cohorts revealed that the mean *PPP1R13B* mRNA expression levels are significantly lower in the intermediate/adverse risk group (*n* = 27) compared to the favorable risk group (*n* = 10, *p* = 0.013), with a mean relative expression level of 0.21 (*PPP1R13B*_int/adv_) vs. 0.46 (*PPP1R13B*_good_), *p* < 0.05, Fig. 1B - whereas the individual levels varied widely (*PPP1R13B*_int/adv_ range 0.03–0.5 vs. *PPP1R13B*_good_ range 0.15–1.4).

Association with the prognostic higher risk groups and the functional aspects of ASPP1 as a proapoptotic protein argues for a direct role of dysfunctional ASPP1 in therapy resistance towards chemotherapy. To address this question, a subanalysis on a fit patient cohort (*n* = 15) undergoing 3 + 7-based therapy was set up to assess whether attenuated levels of *PPP1R13B* mRNA associate with complete remission (CR) status after the first course of induction chemotherapy.

Highly of interest, *PPP1R13B* mRNA expression levels, assessed prior to chemotherapy, were significantly lower in the patient cohort failing complete remission (non-CR) compared to patients who achieved CR (*p* = 0.0015) with a median relative expression level of 0.13 (*PPP1R13B*_nonCR_) vs. 0.51 (*PPP1R13B*_CR_), *p* < 0.01, (Fig. 1C).

Despite relatively low frequency of *TP53* mutations in de novo acute myeloid leukemia – rearrangement of the short arm of chromosome 17 (locating *TP53*) or *TP53* gene mutations are frequently observed in AML with myelodysplasia-related changes and post-cytotoxic changes [19].

Previous reports have argued that deregulation of ASPP may be specifically found in tumors with a *TP53* wildtype (WT) background, such as breast cancer (reviewed by Gasco, Shami and Crook [20]. We therefore asked next, whether deregulation of ASPP1 is limited to de novo AML (with a predominant *TP53* WT background). Comparison of patient cohorts with de novo AML (n = 27) versus patients suffering from AML with myelodysplasia-related changes or post-cytotoxic therapy (*n* = 11) did show attenuated relative *PPP1R13B* mRNA expression levels in both cohorts, however with no significant differences between the groups (median expression 0.29 *PPP1R13B*_denovoAML_ vs. 0.38 *PPP1R13B*_s/tAML_, *p* = 0.77), (Fig. 1D).

To summarize, ASPP1 (*PPP1R13B mRNA)* is frequently attenuated in AML, which associates with a higher risk profile and predicts for therapy failure towards induction chemotherapy in our unselected patient population.

### Validation of attenuated *PPP1R13B* mRNA expression levels in AML using an independent data platform

**Fig. 2:**
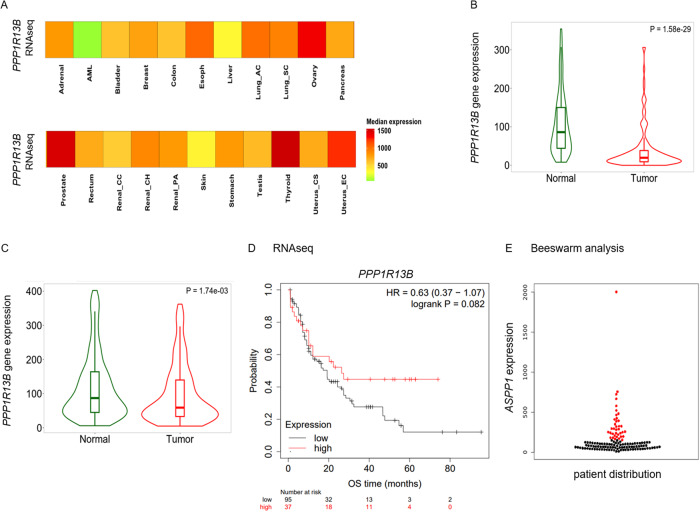
*PPP1R13B* RNAseq expression patterns using the TNMplot data platform [13]. **A** Comparison of 23 tumor entities; heat map provided. **B**/**C** Violin plots to compare *PPP1R13B* expression in healthy myeloid tissue (*n* = 407) vs. AML cohorts (**B**: TARGET, *n* = 145), **C**: TCGA, *n* = 151). Mann–Whitney test. **D** Overall survival (OS) probabilities according to *PPP1R13B* expression. **E** *PPP1R13B* gene expression of the AML patient population visualized in a beeswarm graph.

We next assessed *PPP1R13B* levels in a large pan-cancer RNAseq data platform, which includes 56,938 unique samples from three well-defined datasets (TCGA/TARGET/GTEx) [13], and confirm specifically low *PPP1R13B* expression levels in AML when compared to 22 other tumor entities (Fig. 2A).

Focusing on the acute myeloid leukemia (AML) cohorts within this extensive dataset, the TCGA repository contains a total of 151 AML samples, providing a comprehensive good snapshot of the molecular landscape of AML in the adult population. In parallel, we took an integrative approach by including *n* = 145 pediatric patients with AML drawn from the TARGET program. This two-pronged approach allows for a comprehensive study of AML that spans both the adult and pediatric populations, increasing the depth and applicability of the study.

Together, in this analysis distinct suppression of *PPP1R13B* levels in the pediatric (Fig. 2B) as well as the adult (Fig. 2C) AML cohort was observed. Notably, individual expression levels varied widely, confirming the findings reported in Fig. 1.

Overall survival data were available for the AML cohort of the TCGA repository – which reveals a prognostic advantage for patients harboring high *PPP1R13B* expression levels (Fig. 2D/E), with a median OS of 26.4 months vs. 19.2 months of the *PPP1R13B*_low_ expressors - whereas formal significance was borderline not met due to the small numbers of patients in the cohorts.

### Attenuation of *PPP1R13B* mRNA expression is a prognostic factor in distinct AML subentities

To further validate these observations in a well-defined AML patient cohort, we identified two transcriptomic datasets in the gene expression omnibus (GEO) repository with published follow-up data and mutation status for a selected set of established prognostic genes useful to assess for the correlation between *PPP1R13B* expression levels and survival data (GEO accession numbers GSE1159 [14] and GSE6891 [15].

**Fig. 3:**
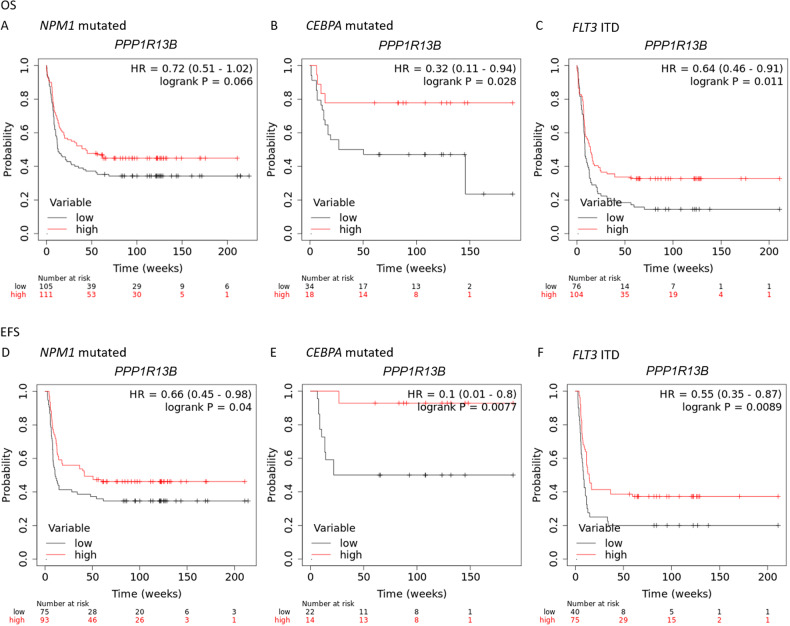
*PPP1R13B* expression and survival in AML. OS (**A**–**C**) and EFS (**D**–**F**) according to mutation status and *PPP1R13B* expression level. (Note: no specific information about allelic [18] or bZip [21] mutation status for *CEBPA* available in the dataset).

Univariate analysis of the entire population did not reveal a significant difference in survival rates according to *PPP1R13B* mRNA levels (*p* = 0.058). However, multivariate analysis of selected prognostically relevant mutations predicting for a good (mut-*NPM1*, mut-*CEBPA*) or adverse outcome (*FLT3* ITD) according to the revised ELN 2022 genetic risk classification [21] reveal that assessment of *PPP1R13B* mRNA expression provides additional prognostic information, which is not reflected by the ELN risk score (Fig. 3A–F).

### *PPP1R13B* knockdown leads to increased proliferation capacity and inhibits chemotherapy induced apoptosis in vitro

**Fig. 4:**
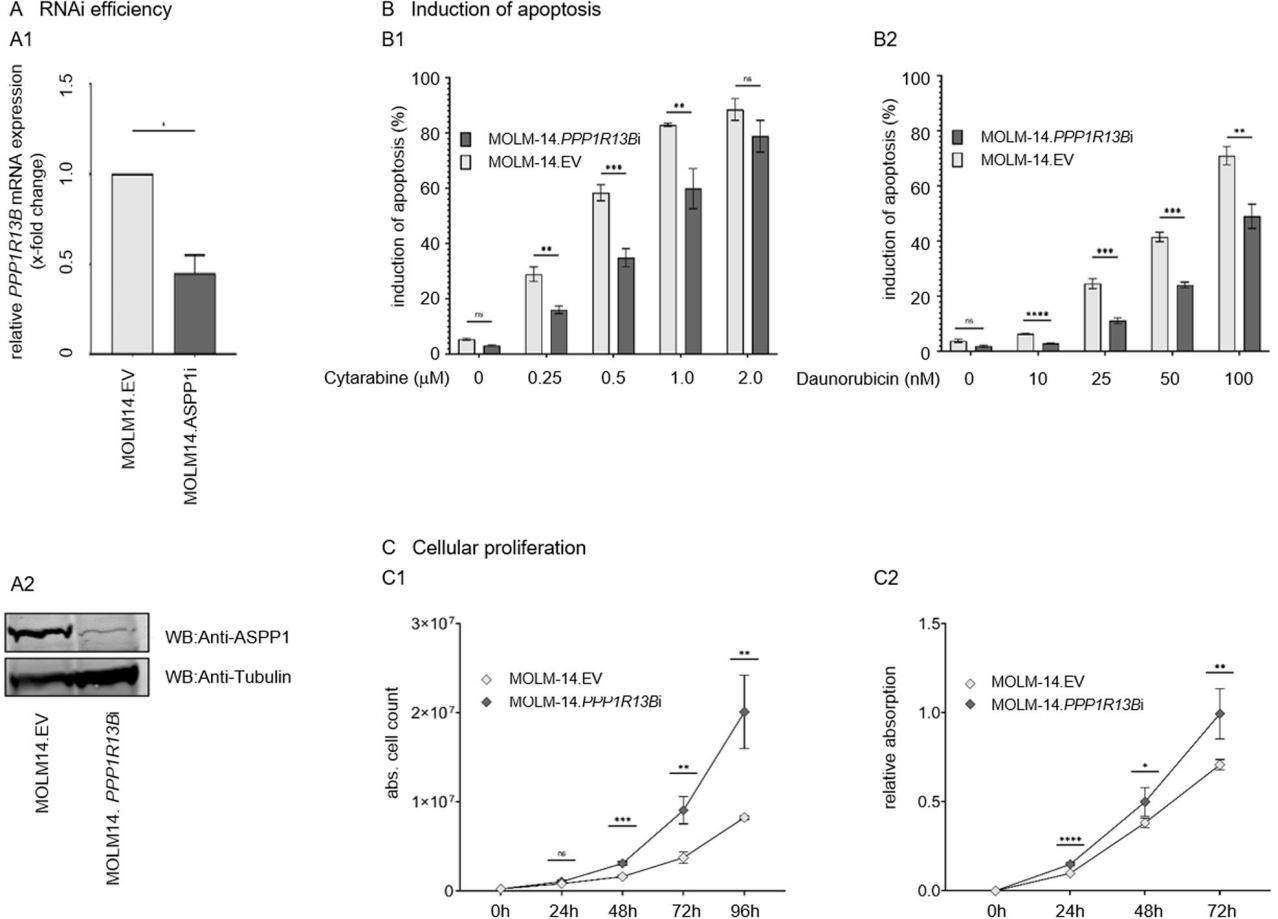
*PPP1R13B* knockdown in a MOLM14 leukemia cell model. **A** Successful RNA-interference in MOLM-14 using an *PPP1R13B*.shRNA lentiviral vector as determined by relative mRNA (A1) and protein (A2) expression levels. **B** Dose-dilution experiments to assess for induction of apoptosis upon cytarabine or daunorubicin exposure in MOLM-14.*PPP1R13B*i vs. MOLM-14.EV cells. **C** Proliferation rates according to cellular doubling times (C1) and an XTT viability assay (C2) of MOLM-14.*PPP1R13B*i vs. MOLM-14.EV cells. EV empty vector, *p < 0.05, **p < 0.01, ***p < 0.001, ****p < 0.0001 (t-test).

To evaluate whether attenuation of ASPP1 expression leads to functional consequences in acute leukemia, we utilized an in vitro *FLT3* ITD *PPP1R13B*-knockdown cell model mimicking the clinical observation of an inferior outcome in this patient population (compare Fig. 3C/F): An RNA-interference (RNAi) approach was used to lentivirally silence *PPP1R13B* in the acute leukemia MOLM-14 cell line. Successful *PPP1R13B* mRNA knockdown and consequently attenuation of ASPP1 protein expression levels was validated by qRT-PCR as well as western immunoblotting (Fig. 4A).

We first addressed, whether attenuation of ASPP1 decreases proapoptotic susceptibility of cells towards chemotherapy. MOLM-14.*PPP1R13B*i or MOLM-14.EV cell strains were treated with standard chemotherapy (daunorubicin or cytarabine as part of the “3 + 7” regimen), in dose-dilution experiments and induction of apoptosis was determined flow cytometrically using an annexin V-based assay.

Indeed, *PPP1R13B*-interference resulted in impaired induction of apoptosis upon treatment with cytarabine or daunorubicin (Fig. 4B). This finding supports our clinical observation that attenuated mRNA expression levels of *PPP1R13B* associate with therapy failure after induction chemotherapy (comp. Fig. 1C, and specifically Fig. 3 for patients with a *FLT3* ITD background).

We next asked whether ASPP1 contributes to aggressiveness of the leukemia phenotype – and assessed cellular proliferation rates of the MOLM14.*PPP1R13B*i model in comparison to the EV cell strains. Cellular doubling times were calculated, which revealed a significant growth advantage for the *PPP1R13B*-interferenced cells (15.2 h vs. 19.0 h (Fig. 4C)).

Increased cellular proliferation rates were further confirmed using an XTT-based assay – which again showed an increased proliferative capacity of the MOLM-14.*PPP1R13B*i cells in comparison to the EV cell strain (Fig. 4C).

### Treatment with DNA methyltransferase inhibitor decitabine results in upregulation of ASPP1 and potentiates chemotherapy induced apoptosis in vitro

**Fig. 5:**
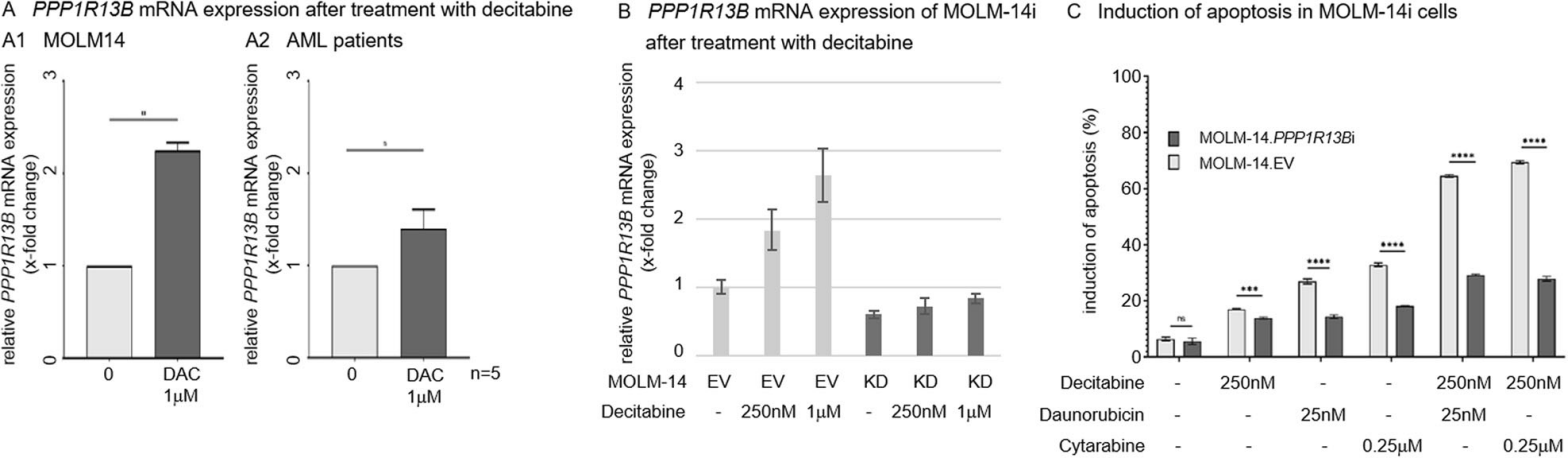
*PPP1R13B* expression in response to HMA. **A** Relative *PPP1R13B* mRNA expression levels (normalized to GAPDH as a house keeping gene) after treatment with decitabine (DAC) as assessed by qRT-PCR in (A1) MOLM14 and (A2) native leukemia blasts (*n* = 5, range 1.03–1.58). **B** Relative *PPP1R13B* expression levels in MOLM-14 *PPP1R13B*-interferenced (MOLM-14i, KD) cells verses empty vector (EV) strains after decitabine treatment. **C** Dose-dilution experiments to assess for induction of apoptosis upon priming with decitabine followed by cytarabine or daunorubicin in MOLM-14.*PPP1R13B*i vs. MOLM-14.EV cells. EV empty vector, *p < 0.05, **p < 0.01, ***p < 0.001, ****p < 0.0001 (t-test).

Previous studies in acute lymphoblastic leukemia linked attenuated expression levels of ASPP1 to hypermethylation of the promoter region [10]. To evaluate, if methylation of the *PPP1R13B* gene promoter is a target of and can be reversed by hypomethylating agents (HMA), MOLM-14 cells and patient derived freshly-isolated AML samples were treated with the DNA methyltransferase inhibitor decitabine. As shown in Fig. 5A, treatment with HMA resulted in significant upregulation of *PPP1R13B* mRNA expression levels in the MOLM-14 cell line as well as 4 out of 5 assessed samples.

Of note, *PPP1R13B* interference of MOLM-14 cells maintains stable suppression of *PPP1R13B* mRNA expression even after treatment with decitabine, while EV strains show upregulation of *PPP1R13B* comparable to the MOLM-14 parental cell line (Fig. 5B).

Consequently, combination of chemotherapy with decitabine resensitized cells towards daunorubicin and cytarabine in an ASPP1-dependent manner as demonstrated in the MOLM-14.*PPP1R13B*i cell line model (Fig. 5C): While, the *PPP1R13B* knockdown strains did not display significant proapoptotic activity – the EV cell strains benefitted from the combination of decitabine and daunorubicin, resp. cytarabine with regard to a significant increase of proapoptotic rates.

## Discussion

Despite significant progress in the understanding of leukemogenesis during the last decade and novel therapeutic options at hand, survival outcomes in AML remain unsatisfactory and the majority of patients still succumb to their disease. A major predictor of survival outcome is response to induction chemotherapy – and failure to achieve a complete remission (CR) results in dismal survival rates [22]. In the referenced study, the vast majority of patients surviving 3 and 5 years achieved a CR during induction or salvage chemotherapy, arguing that CR is a major prognostic parameter - and primary goal of any therapy with curative intention.

One of the major modes of action of chemotherapy is induction of apoptosis as a response to replicative stress and DNA damage [23] – and evading apoptosis is a hallmark of resistance towards chemotherapy [24].

ASPP are a family of proteins binding to key players of cellular homeostasis such as p53 or BCL-2 – whereas interaction with p53 is best investigated and eponymous for the protein family [4]. ASPP1 is preferentially expressed in hematopoietic stem cells (HSC) and together with p53 preserves the genomic integrity of the HSC pool [9].

We now reveal that expression of ASPP1 (*PPP1R13B* mRNA) is frequently attenuated in AML – which has functional consequences leading to inferior survival outcomes: Noticeable, expression patterns of *PPP1R13B* mRNA varied widely in an unselected cohort of patients with newly diagnosed AML. We therefore grouped patients according to the genetic risk profile – und used the European LeukemiaNet (ELN) 2017 genetic risk stratification, which was the current scoring tool when the patients were treated. Interestingly, this analysis revealed that *PPP1R13B* mRNA is frequently decreased in AML – with the lowest mean *PPP1R13B* mRNA levels preferentially detected in higher risk leukemia. However, individual expression levels varied widely and importantly did not associate with a specific ELN risk group. Considering the functional aspects of ASPP1 as an activator of p53-mediated apoptosis [11], this finding suggests, that loss of ASPP1 may result in inferior response rates upon induction chemotherapy. This hypothesis was addressed in a cohort of fit patients undergoing 3 + 7 induction chemotherapy – and strikingly, the non-responder subgroup (i.e. failing complete remission after one induction cycle) had profoundly lower *PPP1R13B* levels when compared to responding patients. Of note, patients with a > 10x fold reduction of *PPP1R13B* expression were exclusively found in the patient cohort failing induction therapy.

To evaluate the significance of attenuated *PPP1R13B* mRNA expression patterns in leukemia, we next performed a database RNAseq screen on a large pan-cancer data platform, which revealed specifically low *PPP1R13B* expression levels in AML when compared to twenty-two other tumor entities. In addition, this screen also allowed comparison of healthy myeloid tissue with leukemia blasts specimens, which unveiled attenuated *PPP1R13B* mRNA levels in the AML cohort.

These observations argue for a dominant role of ASPP1 in leukemogenesis, which is underlined by findings of Yamashita and colleagues, who have demonstrated that ASPP1 together with p53 coordinates the genomic integrity of the hematopoietic stem cell pool – and failure to do so is a first step in oncogenesis of hematopoietic malignancies [9]. In this study deregulation of ASPP1 preferentially lead to development of lymphoid neoplasms, and attenuated levels of ASPP1 was confirmed in native ALL patient samples via methylation of the promoter region [10]. We now reveal in a lentiviral *PPP1R13B* knock-down AML model, that deregulation of ASPP1 directly influences proliferation rates of myeloid leukemia cells, arguing for a contribution to the biology and aggressiveness of myeloid malignancies as well. In this context, *PPP1R13B*-interference lead to impaired susceptibility of leukemic blasts towards chemotherapy in in vitro models, again underlining a central role of ASPP1 to orchestrate induction of apoptosis in response to chemotherapy. Consequently, low *PPP1R13B* mRNA expression levels predicted for inferior survival rates in the above mentioned RNAseq screen adult AML cohort (TCGA).

To further confirm this observation in a larger independent patient cohort, a transcriptomic dataset of 1105 patients (GSE1159/GSE6891) treated with intensive chemotherapy in well-defined clinical trials, was identified. While *PPP1R13B* mRNA expression levels failed to predict survival outcomes of the entire patient population, multivariate analysis of genetic subgroups revealed partially impressive differences in survival rates depending on the relative *PPP1R13B* expression status within the respective subcohort assessed. This may have direct clinical importance, as assessment of *PPP1R13B* mRNA expression levels provide additional information, which is not reflected by the ELN risk score: In a multivariate analysis of frequent mutations detected in AML, low mRNA levels of *PPP1R13B* predicted for a higher chance of therapy failure - and these patients may benefit from early therapy intensification strategies, which may include addition of novel agents such as BCL-2 BH3 mimetics (venetoclax, tested in an ongoing trial (NCT04628026) or early allogeneic stem cell transplantation (Stelljes et al., Blood (2022) 140 (Supplement 1): [9–11].

It has to be noted that not all assessed subentities displayed a survival benefit of the ASPP1^high^-expressor cohort – but we observed opposite effects for some entities such as core binding factor (CBF) leukemia. While these observations are somewhat confusing and contrary to the well described proapoptotic functions of ASPP1, this may explain the non-significant, survival correlation found for the entire population (Supplementary Fig. S1). Even though we are not able to provide a definite explanation for this observation at this point, several scenarios are conceivable. We have provided evidence from the co-family member ASPP2 that dominant-negative splicing isoforms exist [25]. Homology considerations suggest that this is likely the case for *PPP1R13B* as well. Indeed, altered splicing of *PPP1R13B* has just recently been described [26] and this might play a predominant role in distinct molecular subgroups in AML as well. In line, a transcriptome analysis in pediatric CBF AML has revealed distinct alternative splicing events which are differentially expressed in the respective subgroups [27].

Further, it has been suggested that depending on subcellular localization and binding partners, ASPP1 may either mediate nuclear pro-apoptotic but also cytoplasmatic anti-apoptotic functions [12] – and C-terminal splicing may enhance cytoplasmatic ASPP1 as we have reported for alternatively spliced ASPP2κ [25]. However, these fascinating aspects need further confirmation.

Comparing the survival data of the assessed patient populations including the transcriptome and the RNAseq set, it is obvious that survival data of unselected patient populations may differ. This may depend on the molecular background, the respective therapy and fitness as well as technical issues including expression thresholds. These aspects will need to be taken into account when proceeding to develop ASPP1 as a prognostic/predictive marker in AML.

The *PPP1R13B* promoter is methylated in ALL and hypomethylating agents (HMA) reconstitute ASPP1 levels [10]. Similar mechanisms are suggested for myeloid leukemias – and HMA may therefore provide a tool to re-sensitize leukemic blasts towards (chemo)therapy.

Here, we now show that HMA are capable to increase *PPP1R13B* mRNA expression levels in in vitro as well as ex vivo native AML blasts, and this leads to increased apoptosis rates when combined with induction chemotherapy. Considering that HMA such as decitabine are non-specific and cause hypomethylation of a myriad of gene promoters, it is all the more remarkable that HMA-mediated upregulation of ASPP1 alone is sufficient to significantly affect susceptibility to chemotherapy, as we confirm in our *PPP1R13B*-interference model. This observation puts ASPP1 into a central role to control apoptosis and provides a rationale for further testing HMA+Cx combinations in patients at risk to fail therapy. Notably, HMA plus induction chemotherapy strategies have been followed in an earlier trial [28], which did not reveal a benefit of this combination compared to standard therapy in upfront strategies. However, in this trial cytarabine was omitted in the HMA arms due to safety concerns – whereas we show a super/additive effect of this combination in our model. Second, relevant genetic subgroups were excluded (i.e. mutant-*NPM1* and *FLT3* ITD leukemias), which may specifically benefit from such an approach as suggested by our multivariate analysis.

Consequently, it still remains to be elucidated whether defined high risk patient subgroups (i.e. *PPP1R13B*_low_ expressors) may benefit from a salvage strategy including HMA as an adjunct to standard therapy.

To conclude, our results demonstrate that dysfunctional regulation of ASPP1 (*PPP1R13B* mRNA) expression is frequently observed in AML, which associates with inferior therapy responses and survival outcomes. Prospective clinical studies are warranted to evaluate the role of ASPP1 (*PPP1R13B*) as a biomarker for risk stratification, which is not reflected by ELN genetic risk stratification. Further, we provide a rationale to re-sensitize high-risk *PPP1R13B*_low_ patients towards chemotherapy using HMA priming - and this strategy should be elucidated in future trials.

### Supplementary information

Supplemental Figure S1

Original Immunoblot acc. Fig. 4A2

## Data Availability

All datasets generated and analyzed during this study are included in this published article and its Supplementary Information files. Additional data are available from the corresponding author on reasonable request.
