# Supplementary figures and images for: Evaluation of apoptosis stimulating protein of TP53-1 (ASPP1/PPP1R13B) to predict therapy resistance and overall survival in acute myeloid leukemia (AML)
Source: Cell Death Dis. 2024 Jan 10;15(1):25. doi: 10.1038/s41419-023-06372-0 (PMC10776670; doi:10.1038/s41419-023-06372-0)

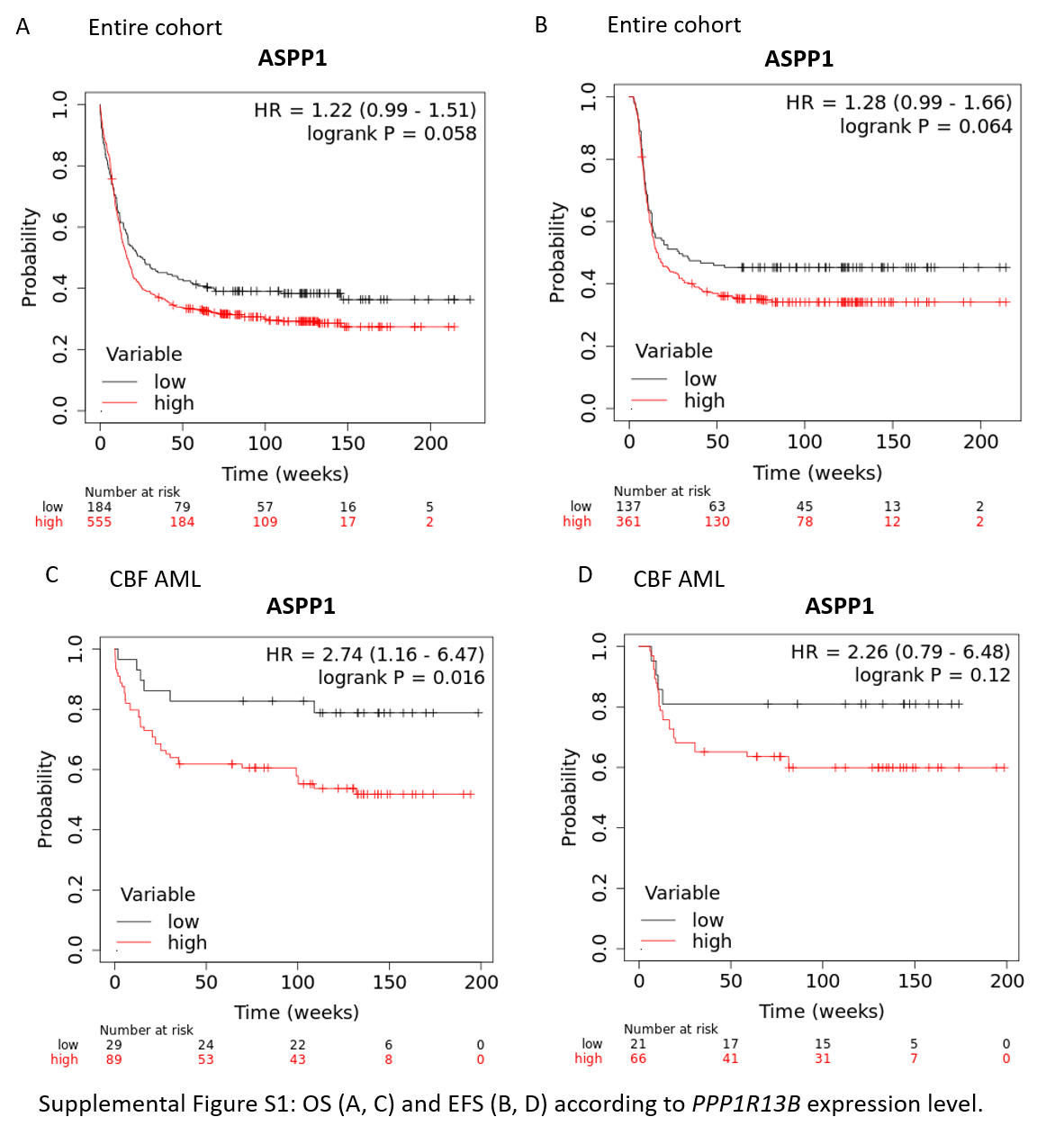

Supplement: Supplementary file 1 — Supplemental Figure S1 [file 41419_2023_6372_MOESM1_ESM.tif]

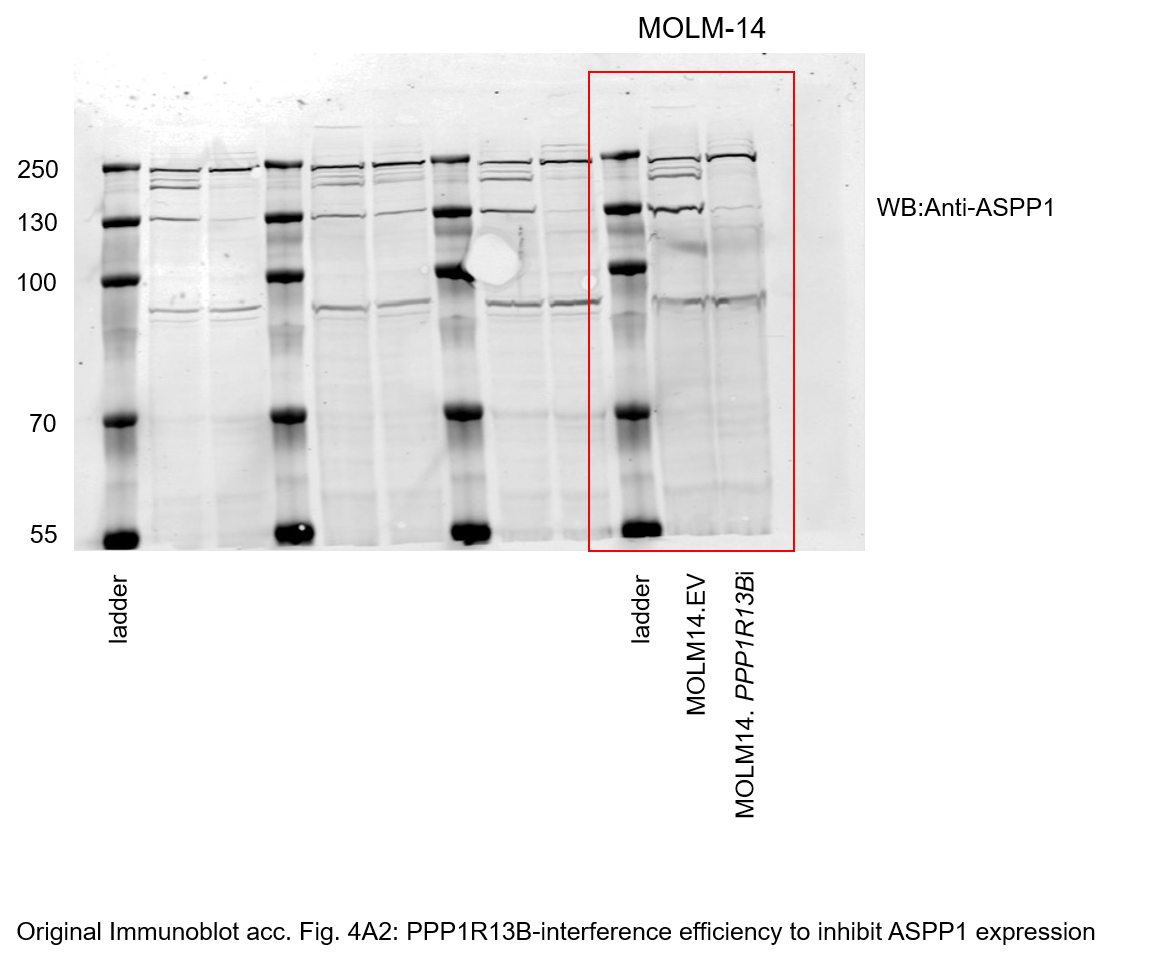

Supplement: Supplementary file 2 — Original Immunoblot acc. Fig. 4A2 [file 41419_2023_6372_MOESM2_ESM.tif]
